# Associations of composite dietary antioxidant index with suicidal ideation incidence and mortality among the U.S. population

**DOI:** 10.3389/fnut.2024.1457244

**Published:** 2024-10-07

**Authors:** Shaoqun Huang, Weimin Zhao, Seok Choi, Hongyang Gong

**Affiliations:** ^1^Department of Oncology Surgery, Fuzhou Hospital of Traditional Chinese Medicine Affiliated to Fujian University of Traditional Chinese Medicine, Fuzhou, Fujian, China; ^2^Department of Clinical Medicine, School of Medicine, Shihezi University, Shihezi, China; ^3^Department of Physiology, College of Medicine, Chosun University, Gwangju, Republic of Korea

**Keywords:** CDAI, suicidal ideation, all-cause mortality, NHANES, mortality

## Abstract

**Background:**

The relationship between CDAI and suicidal ideation is unclear. This study investigates the relationship between CDAI and suicidal ideation and examines the association between CDAI and all-cause mortality (ACM) or cardiovascular disease mortality (CVM) among participants with and without suicidal ideation.

**Methods:**

Data from seven NHANES cycles (2005–2018) were analyzed using cross-sectional and prospective cohort studies. Weighted multivariable logistic regression models, restricted cubic spline (RCS) plots, and subgroup analyses explored the association between CDAI and suicidal ideation. Kaplan–Meier (KM) curves, weighted multivariable Cox proportional hazards models, and RCS assessed the relationship between CDAI and CVM or ACM.

**Results:**

Among 30,976 participants aged over 20, 1,154 (3.72%) had suicidal ideation. Higher CDAI levels (Quartile 4) were associated with a 28% reduction in suicidal ideation compared to lower levels (Quartile 1). Over an average follow-up of 89 months, 3,267 participants (7.6%) died, including 808 (1.8%) from cardiovascular causes. Higher CDAI levels were linked to a 30, 68, and 28% reduction in ACM in the total population, those with suicidal ideation, and those without, respectively. CVM was reduced by 40% in the total population and by 41% in those without suicidal ideation.

**Conclusion:**

CDAI is negatively associated with suicidal ideation and correlated with reduced ACM and CVM among participants with and without suicidal ideation.

## 1 Introduction

Suicide stands as a significant global concern in both public health and society, ranking as the second highest cause of mortality among individuals aged 15 to 29 (1). Gustavo Turecki and colleagues reported a suicide rate of nearly 11.4 per 100,000 individuals, equating to roughly 1 million global suicides annually (2). The ramifications of suicide extend far beyond the individual, affecting families, communities, and even nations, leaving lasting emotional and societal scars (3). Suicidal ideation, as an early psychological activity preceding suicide, is considered a strong predictor of suicidal behavior (4). Therefore, identifying modifiable factors linked to suicidal thoughts at an early stage is imperative.

CDAI is a simple, cost-effective measure for evaluating an individual’s intake of dietary antioxidants. It has been associated with various chronic diseases, such as coronary heart disease (5), stroke (6), chronic respiratory diseases (7), and hyperlipidemia (8). Additionally, research by Juanjuan Luo and Xiying Xu has shown that CDAI is linearly and negatively associated with depression (OR = 0.77, 95% CI [0.67, 0.89]) (9). Roughly 90% of individuals experiencing suicidal thoughts have been reported to contend with mental disorders like depression (10). Therefore, we hypothesize that CDAI is negatively correlated with the odds of suicidal ideation.

Therefore, this study seeks to explore the correlation between CDAI and suicidal thoughts, as well as assess the connection between CDAI ACM and CVM in both individuals with and without suicidal ideation. The study population is derived from data spanning seven cycles of the NHANES from 2005 to 2018.

## 2 Methods

### 2.1 Study participants

The NHANES is an ongoing, stratified, multistage sampling program designed to assess the health and nutritional status of adults and children in the United States. It encompasses a variety of health outcomes and nutritional indicators (11). The NHANES study has been approved by the Research Ethics Review Board of the NCHS, and all participants provided written informed consent (12).

Among the 70,190 participants in the seven NHANES cycles from 2005 to 2018, 39,749 were aged 20 years or older. Following the exclusion of participants with incomplete CDAI data (*n* = 6,739), missing suicidal ideation data (*n* = 1,980), and missing follow-up data (*n* = 54), the study comprised a total of 30,976 participants (Supplementary Figure S1).

### 2.2 Exposure and outcome variables

The main exposure variable in this study is CDAI. Based on previous research (9, 13), six dietary minerals and vitamins (manganese, selenium, zinc, and vitamins A, C, and E) were used to calculate CDAI for all participants. The detailed calculation formula is provided in Supplementary Table S1. The primary outcomes were suicidal ideation and mortality (ACM and CVM). Suicidal ideation was assessed using the PHQ-9. Data on ACM and CVM were obtained from the National Death Index (NDI) up to December 31, 2019.^1^ Specific definitions of these variables are provided in Supplementary Table S1.

### 2.3 Covariables

According to previous studies (14, 15), the covariates considered in the study encompass age, sex, race, marital status, education level, PIR, obesity, smoking, alcohol consumption, hypertension, and diabetes. For comprehensive details on these covariates, kindly consult Supplementary Table S1.

### 2.4 Statistical analysis

Sampling weights were used in all statistical analyses to ensure that the estimated data were nationally representative. In our study, the two-day dietary sample weight (WTDR2D) was used as the weighting variable, with the new weights (for 2005–2018) calculated as 1/7 × WTDR2D. Baseline characteristics were stratified into two groups based on the presence or absence of suicidal ideation and into quartiles based on CDAI. Continuous variables are expressed as mean (SD), whereas categorical variables are depicted as frequencies (9). Differences between the non-suicidal ideation group and the suicidal ideation group were assessed using weighted *t*-tests for continuous variables and weighted chi-square tests for categorical variables (9).

Weighted logistic regression was used to investigate the correlation between CDAI and suicidal ideation. Kaplan–Meier curves were utilized to depict mortality rates among different quartiles of CDAI, with comparisons conducted via log-rank tests. Weighted Cox proportional hazard models were employed to examine the association between CDAI and mortality rates among participants, stratified by the presence or absence of suicidal ideation. Three logistic regression models and three Cox proportional hazards models were constructed: In model 1, no adjustment was made for any confounding factors. Model 2 was adjusted for age, sex, race, marital status, education level, and PIR. Model 3 additionally accounted for obesity, smoking status, alcohol status, hypertension, and diabetes. Additionally, in Model 3, CDAI was treated as a continuous variable, and RCS curves were employed to elucidate the dose–response relationship between CDAI and the odds of suicidal ideation or all-cause mortality.

To further explore the relationships mentioned above, we conducted subgroup analyses by the variables in Model 3. Interaction analyses were performed to examine whether there were differential associations between subgroups. Statistical analyses were performed using R software (version 4.3.1). A significance level of *p* < 0.05 (two-sided) was considered statistically significant.

## 3 Result

### 3.1 Baseline characteristics

In this study, a total of 30,976 participants aged 20 years or older were included, consisting of 15,051 males and 15,925 females. Baseline characteristics of participants with and without suicidal ideation are shown in Table 1. The mean (SD) CDAI was 1.28 (4.83), and the prevalence of suicidal ideation was 3.2% (equivalent to 5.92 million of the U.S. population). Over an average follow-up period of 89 months, a total of 3,267 participants (7.6%) died from all causes, with 808 (1.8%) deaths attributed to cardiovascular causes. Preliminary assessment revealed that a larger proportion of participants with suicidal ideation were female, younger in age, White, unmarried, of higher socioeconomic status, and current or former smokers. Additionally, participants with suicidal ideation were more likely to have lower CDAI. Baseline characteristics of participants stratified by the presence or absence of suicidal ideation based on CDAI are presented in Supplementary Tables S2–S4.

**Table 1:** The baseline characteristics of all participants were stratified by suicidal ideation.

Characteristic	Overall, *N* = 30,976 (100%)	Non- suicidal ideation, *N* = 29,822 (97%)	Suicidal ideation, *N* = 1,154 (3.2%)	*p* value
**Age (%)**				0.058
*20–40*	10,811 (37%)	10,451 (37%)	360 (35%)	
*41–60*	10,290 (38%)	9,830 (38%)	460 (42%)	
*>60*	9,875 (25%)	9,541 (25%)	334 (23%)	
**Gender (%)**				0.214
*Male*	15,051 (48%)	14,529 (48%)	522 (46%)	
*Female*	15,925 (52%)	15,293 (52%)	632 (54%)	
**Race (%)**				**<0.001**
*Non-Hispanic White*	13,614 (69%)	13,157 (69%)	457 (61%)	
*Non-Hispanic Black*	6,659 (11%)	6,438 (11%)	221 (12%)	
*Other*	5,952 (12%)	5,673 (12%)	279 (18%)	
*Mexican American*	4,751 (8.1%)	4,554 (8.0%)	197 (9.3%)	
**Married/live with partner (%)**				**<0.001**
*No*	12,334 (36%)	11,696 (35%)	638 (53%)	
*Yes*	18,642 (64%)	18,126 (65%)	516 (47%)	
**Education level (%)**				**<0.001**
*Below high school*	7,247 (15%)	6,804 (14%)	443 (27%)	
*High school or above*	23,729 (85%)	23,018 (86%)	711 (73%)	
**PIR (%)**				**<0.001**
*Not Poor*	19,850 (80%)	19,326 (81%)	524 (60%)	
*Poor*	8,672 (20%)	8,140 (19%)	532 (40%)	
**Obesity (%)**				**0.006**
*No*	18,774 (62%)	18,133 (62%)	641 (57%)	
*Yes*	11,937 (38%)	11,445 (38%)	492 (43%)	
**Smoking (%)**				**<0.001**
*Never*	17,042 (55%)	16,547 (55%)	495 (44%)	
*Former*	7,697 (25%)	7,417 (25%)	280 (24%)	
*Current*	6,237 (20%)	5,858 (19%)	379 (33%)	
**Drinking (%)**				**<0.001**
*Former*	4,973 (13%)	4,728 (13%)	245 (19%)	
*Heavy*	5,950 (21%)	5,668 (21%)	282 (26%)	
*Mild*	10,157 (37%)	9,883 (37%)	274 (28%)	
*Moderate*	4,684 (18%)	4,532 (18%)	152 (15%)	
*Never*	4,256 (11%)	4,100 (11%)	156 (12%)	
**Hypertension (%)**				**0.001**
*No*	17,298 (61%)	16,738 (62%)	560 (55%)	
*Yes*	13,347 (39%)	12,768 (38%)	579 (45%)	
**Diabetes (%)**				**<0.001**
*No*	11,632 (75%)	11,254 (75%)	378 (67%)	
*Yes*	5,477 (25%)	5,197 (25%)	280 (33%)	
**Depression, *n* (%)**	2,814 (7.8%)	2,035 (5.9%)	779 (64%)	**<0.001**
**All-cause mortality, *n* (%)**	3,267 (7.6%)	3,091 (7.5%)	176 (12%)	**<0.001**
**Cardiovascular disease mortality, *n* (%)**	808 (1.8%)	765 (1.8%)	43 (2.6%)	0.058
**CDAI (mean (SD))**	1.28 (4.83)	1.31 (4.82)	0.40 (5.13)	**<0.001**

Mean (SD) for continuous variables: the *p* value was calculated by the weighted linear regression model. Percentages (weighted *N*, %) for categorical variables: the *p* value was calculated by the weighted chi-square test. CDAI, composite dietary antioxidant index; PIR, poverty income ratio.

### 3.2 Association of CDAI and suicidal ideation

**Figure 1:**
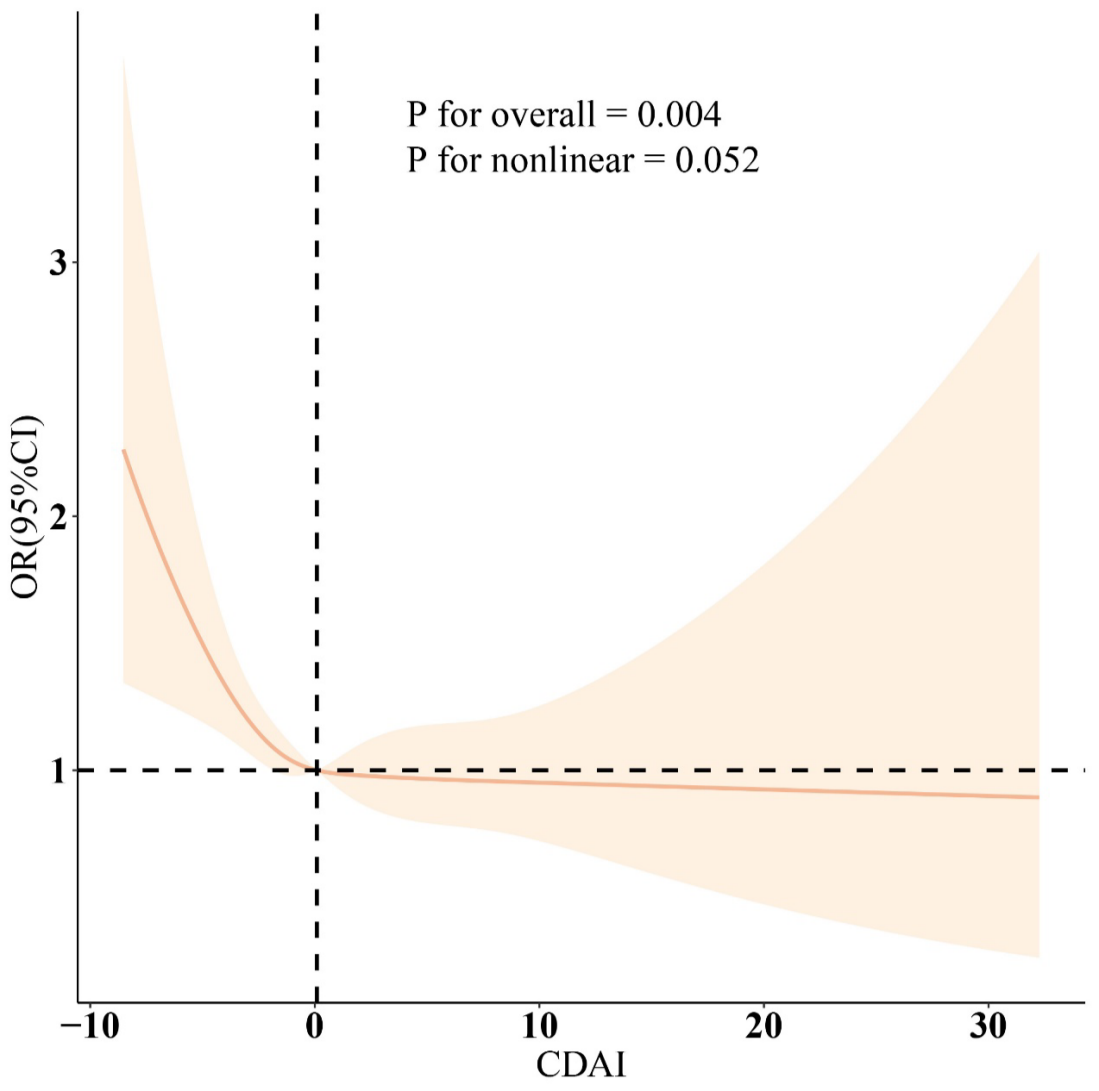
The smooth curve fitting analysis of CDAI and suicidal ideation. OR (solid lines) and 95% confidence levels (shaded areas) were adjusted for age, gender, education level, marital status, PIR, race, obesity, smoking, drinking, hypertension, and diabetes. PIR, poverty income ratio; CDAI, composite dietary antioxidant index; OR, odds ratio; CI, confidence interval.

**Figure 2:**
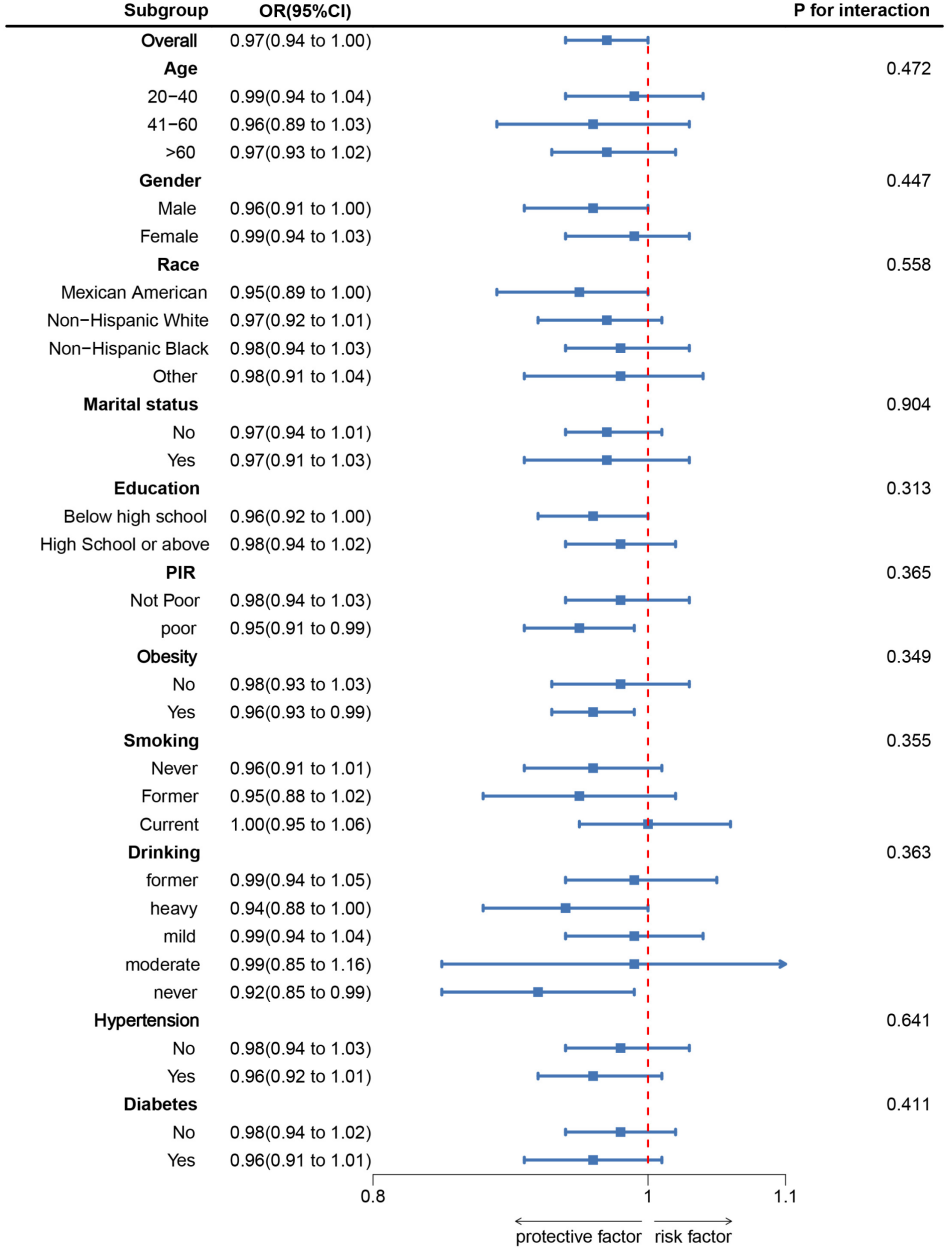
Subgroup analysis between CDAI and suicidal ideation. ORs were calculated as each unit increased in CDAI. Analyses were adjusted for age, gender, education level, marital status, PIR, race, obesity, smoking, drinking, hypertension, and diabetes. PIR, poverty income ratio; CDAI, composite dietary antioxidant index; OR, odds ratio; CI, confidence interval.

As illustrated in Table 2, three distinct models were employed to evaluate the relationship between CDAI and suicidal ideation. In the model adjusting for all covariates, an increase of 1 unit in CDAI was associated with a 3% decrease in the odds of suicidal ideation (OR: 0.97, 95% CI: 0.94, 1.00). Furthermore, compared to the lowest quartile (Q1) of CDAI, the odds of suicidal ideation in the highest quartile (Q4) were 0.72 (95% CI, 0.53, 0.98). RCS analysis (Figure 1) further demonstrated a significant negative correlation between CDAI and the odds of suicidal ideation. The results of the subgroup analysis (Figure 2) also confirmed the consistency of the findings.

**Table 2:** ORs (95% CIs) for suicidal ideation according to the CDAI.

Characteristics	Model 1 [OR (95% CI)]	*p-value*	Model 2 [OR (95% CI)]	*p*-value	Model 3 [OR (95% CI)]	*p*-value
Continuous	0.95 (0.93, 0.98)	<0.001	0.97 (0.95, 0.99)	0.012	0.97 (0.94, 1.00)	0.042
Quartile						
Q1	1 (ref.)		1 (ref.)		1 (ref.)	
Q2	0.58 (0.46, 0.74)	<0.001	0.69 (0.54, 0.90)	0.006	0.80 (0.58, 1.09)	0.200
Q3	0.52 (0.42, 0.65)	<0.001	0.65 (0.51, 0.82)	<0.001	0.78 (0.58, 1.06)	0.110
Q4	0.56 (0.44, 0.70)	<0.001	0.68 (0.52, 0.88)	0.003	0.72 (0.53, 0.98)	0.037
*P for trend*	<0.001		0.001		0.041	

Model 1: no covariates were adjusted.

Model 2: age, gender, education level, marital, PIR, and race were adjusted.

Model 3: age, gender, education level, marital status, PIR, race, obesity, smoking, drinking, hypertension, and diabetes were adjusted.

PIR, poverty income ratio; CDAI, composite dietary antioxidant index; OR, odds ratio; CI, confidence interval.

### 3.3 Correlation between CDAI and mortality in participants with or without suicidal ideation

**Figure 3:**
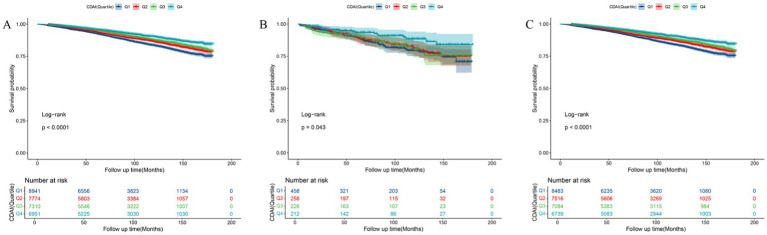
Kaplan–Meier analysis of all-cause mortality in **(A)** total participants, **(B)** participants with suicidal ideation, and **(C)** participants without suicidal ideation.

Among all participants, 3,267 individuals died. K-M survival curves showed that higher CDAI was correlated with lower ACM in all participants, regardless of their suicidal ideation status (all *p*-values <0.05, as shown in Figure 3). Multivariable Cox regression models in Table 3 indicated that compared to participants with lower CDAI, those with higher CDAI had a 30% reduction in ACM (HR = 0.70, 95% CI: 0.60, 0.83), a 68% reduction in ACM among participants with suicidal ideation (HR = 0.32, 95% CI: 0.13, 0.78), and a 28% reduction in ACM among participants without suicidal ideation (HR = 0.72, 95% CI: 0.61, 0.85). CVM decreased by 40% in the total population (HR = 0.60, 95% CI: 0.42, 0.85) and by 41% in participants without suicidal ideation (HR = 0.59, 95% CI: 0.41, 0.86). RCS analysis (Supplementary Figure S2) and subgroup analyses also supported these findings (Supplementary Figures S3–S5).

**Table 3:** HRs (95% CIs) for all-cause mortality and cardiovascular mortality according to the CDAI.

Characteristics	All-cause mortality (ACM) [HR (95% CI)]^a^	*p*-value	Cardiovascular mortality (CVM) [HR (95% CI)]^a^	*p*-value
**All participants**				
Continuous	0.97 (0.96, 0.98)	<0.001	0.96 (0.93, 0.99)	0.005
Quartile				
Q1	1 (ref.)		1 (ref.)	
Q2	0.89 (0.77, 1.02)	0.094	0.86 (0.65, 1.12)	0.300
Q3	0.77 (0.64, 0.91)	0.003	0.78 (0.58, 1.06)	0.120
Q4	0.70 (0.60, 0.83)	<0.001	0.60 (0.42, 0.85)	0.005
*P for trend*	<0.001		0.004	
**Suicidal ideation**				
Continuous	0.95 (0.88, 1.03)	0.200	0.96 (0.85, 1.08)	0.500
Quartile				
Q1	1 (ref.)		1 (ref.)	
Q2	0.85 (0.46, 1.57)	0.600	0.13 (0.03, 0.54)	0.005
Q3	1.19 (0.59, 2.41)	0.600	0.61 (0.13, 2.91)	0.500
Q4	0.32 (0.13, 0.78)	0.013	0.34 (0.05, 2.16)	0.300
*P for trend*	0.025		0.600	
**Non-suicidal ideation**				
Continuous	0.97 (0.96, 0.99)	<0.001	0.96 (0.93, 0.99)	0.005
Quartile				
Q1	1 (ref.)		1 (ref.)	
Q2	0.89 (0.76, 1.04)	0.140	0.88 (0.66, 1.17)	0.400
Q3	0.76 (0.63, 0.91)	0.003	0.77 (0.56, 1.08)	0.130
Q4	0.72 (0.61, 0.85)	<0.001	0.59 (0.41, 0.86)	0.006
*P for trend*	<0.001		0.004	

a Age, gender, education level, marital status, PIR, race, obesity, smoking, drinking, hypertension, and diabetes were adjusted.

PIR, poverty income ratio; CDAI, composite dietary antioxidant index; HR, hazard ratio; CI, confidence interval.

## 4 Discussion

In this nationally representative sample study of American adults, a notable negative correlation was observed between CDAI and the likelihood of experiencing suicidal ideation. Furthermore, it is noteworthy that higher CDAI was correlated with reduced mortality in both participants with and without suicidal ideation. These findings underscore the potential impact of CDAI on the incidence of suicidal ideation and mortality rates in these patients, emphasizing the importance of dietary antioxidants in monitoring and reducing the incidence of suicidal ideation and mortality rates.

To the best of our knowledge, this study represents the first examination of the relationship between CDAI and the prevalence of suicidal ideation. In recent years, there has been increasing scholarly attention to the role of dietary antioxidants in suicidal ideation. Findings from a randomized placebo-controlled clinical trial by Sahraian et al. (16) suggest that vitamin C may serve as an adjunctive agent in the treatment of suicidal behaviors. A study by Strumila et al. (17) indicates that patients with low selenium levels have a higher risk of suicide, accompanied by a more frequent history of suicide attempts. Additionally, research by Sher (18) suggests that selenium deficiency plays a significant role in the pathophysiology of suicidal behavior in alcohol abusers.

In a cross-sectional study based on a national survey involving 4,561 American participants, Dong Huang et al. found (14) that, after adjusting for potential confounding factors, the second quartile (compared to the highest quartile) of serum zinc levels had a higher risk of suicidal ideation [OR = 2.63; 95% CI: (1.53, 4.53)]. In this study, we observed similar results when applying the CDAI calculated based on manganese, selenium, zinc, and vitamins A, C, and E, indicating a negative association between CDAI and the odds of suicidal ideation. Furthermore, CDAI was also negatively correlated with the mortality rate in the population with suicidal ideation.

Although the mechanisms underlying the negative relationship between CDAI and the prevalence and mortality of suicidal ideation are extensive and complex, some studies have provided potential molecular mechanisms for these associations, primarily oxidative stress. Research by Koweszko et al. (19) found that levels of oxidative stress biomarkers (NADPH oxidase, advanced oxidative protein products, and oxidative stress index) were significantly higher in subjects with a history of suicidal ideation in the past three months. Additionally, a study by Loo et al. (20) demonstrated that compared to the group without suicidal ideation, those with a lifetime history of suicidal ideation had higher levels of oxidative stress (i.e., DNA damage). DNA damage can lead to dysfunction in DNA repair mechanisms, subsequently resulting in abnormal neurotransmission, impaired neuroplasticity, and dysfunctional energy metabolism in the brain (21). The consumption of foods abundant in antioxidants can mitigate oxidative stress, safeguarding cells and tissues from damage induced by free radicals. This may consequently reduce the occurrence of suicidal ideation (14, 16). Based on previous research, antioxidants such as vitamins C and E have been shown to counteract oxidative damage, protecting cell membranes from harm caused by free radicals, thereby reducing the risk of various chronic diseases (such as diabetes (22), cardiovascular diseases (23, 24), etc.) and lowering the risk of ACM and CHD death in the elderly (25).

Furthermore, chronic inflammation is another factor. Research by Lee et al. (26) suggests that brain inflammation can promote the occurrence of depression, a condition often accompanied by suicidal ideation (27). A diet rich in antioxidants can reduce neuroinflammation by modulating various inflammatory factors. Studies by Xu et al. (28) indicate that dietary vitamins A and E, along with the trace element zinc, can lower levels of various inflammatory factors (such as IL-6, TNF-*α*, and IFN-*γ*), thus mitigating the impact of brain inflammation on depression. Additionally, chronic inflammation is associated with the occurrence of various diseases (such as hypertension, diabetes, kidney disease, etc. (29–31)) and increased risk of mortality (32, 33). Consuming antioxidant-rich foods can reduce inflammatory factors and lower the risk of mortality (9).

Thirdly, improving gut microbiota is crucial. Research indicates that dysbiosis of gut microbiota is associated with depression (34) and poor prognosis for various chronic diseases (35, 36). A study by Ogdur et al. (37) revealed significant differences in the gut microbiota composition of suicide victims compared to controls, particularly in genera such as Bacteroides, Clostridium, Lactobacillus, and Bifidobacterium. Therefore, supplementing probiotics to individuals with gut microbiota imbalance may help reduce the risk of suicide death. Moreover, consuming antioxidant-rich foods can regulate gut microbiota to alleviate depressive symptoms and prolong patients’ lives (38, 39).

The findings from this study could provide valuable insights for the management and prevention of suicide risk. Firstly, this study represents the pioneering use of CDAI in predicting the risk of suicidal ideation, thus holding substantial clinical significance. Secondly, we meticulously considered appropriate sampling weights in our analysis to mitigate biases stemming from oversampling, thereby enhancing the reliability of our conclusions. Lastly, due to the nationally representative sample of American adults, these findings can be extrapolated to a wider population (40). However, the study also has several limitations: (1) The cross-sectional design hinders establishing causality, thus the causal relationship between CDAI and suicidal ideation cannot be determined. (2) Diagnosis of suicidal ideation primarily relies on questionnaire formats, which may entail measurement errors. (3) Despite adjustments for many other confounding factors, limitations of the NHANES database prevent the complete elimination of all potential confounders’ final impact on the study outcomes (40). (4) It is important to acknowledge that factors such as treatment for suicidal patients, experiences with substance addiction, and the presence of gastrointestinal disorders or food allergies may influence the results of this study. Future research should account for these factors and their potentially confounding effects to provide a more comprehensive understanding of the relationship between CDAI and suicidal ideation. (5) Furthermore, individuals frequently use psychotropic medications and counseling interventions to prevent or alleviate psychological symptoms without a doctor’s prescription. These factors may influence the study’s results, and we urge a careful interpretation of the findings.

## 5 Conclusion

In conclusion, our study indicates a negative relationship between CDAI and the occurrence of suicidal ideation as well as all-cause mortality. These findings may aid public health officials and mental health professionals in formulating relevant policies to prevent the occurrence of suicide tragedies.

## Data Availability

The datasets presented in this study can be found in online repositories. The names of the repository/repositories and accession number(s) can be found below: Publicly available datasets were analyzed in this study. The data can be found here: https://www.cdc.gov/nchs/nhanes/.

## Footnotes

^1^ https://www.cdc.gov/nchs/data-linkage/mortality.htm
