# Supplementary material for: Associations of composite dietary antioxidant index with suicidal ideation incidence and mortality among the U.S. population
Source: Front Nutr. 2024 Oct 7;11:1457244. doi: 10.3389/fnut.2024.1457244 (PMC11492068; doi:10.3389/fnut.2024.1457244)
Supplement: Supplementary file 1 [file Data_Sheet_1.docx]

**Supplementary Material**

**Table S1.** Definition of variables involved in this study.

| Variables | Description in NHANES |
| --- | --- |
| CDAI | CDAI was determined by consuming manganese, selenium, zinc, and vitamins A, C, and E six antioxidants from dietary data. Dietary assessments were based on two 24-hour recall interviews conducted by trained dietary interviewers. The first recall was carried out in person using a standardized protocol during the medical examination at the mobile screening center. The second recall was conducted by telephone within 3 to 10 days of the first recall. Nutritional intakes were calculated based on food intake through the use of a revised nutritional database which translates each individual's food intake into nutrients. The detailed calculation formula was as follows:  $\boldsymbol{C}\boldsymbol{DAI=}\sum_{\boldsymbol{i=1}}^{\boldsymbol{n=6}} \frac{\boldsymbol{In}\boldsymbol{dividual Intake-Mean}}{\mathbf{S}\boldsymbol{D}}$ |
| Suicidal ideation | PHQ-9 is widely used to evaluate individuals' mental health during the last 2 weeks, and is administered as part of the NHANES. The scale consists of nine items, each of which is scored as follows: 0 = not at all; 1 = several days; 2 = more than half the days; and 3 = nearly every day (with an overall score ranging from 0 to 27).  Suicidal ideation was assessed using the response to Item #9 of PHQ-9: “Over the last 2 weeks, how often have you been bothered by the following problem: Thoughts that you would be better off dead or of hurting yourself in some way?”. A score of 1–3 was defined as “suicidal ideation,” and a score of 0 was defined as “non-suicidal ideation”. |
| Mortality: all-cause mortality (ACM) and cardiovascular disease mortality (CVM) | ACM and CVM were determined by linking study data to the National Mortality Index through December 2019 (https://www.cdc.gov/nchs/data-linkage/mortality.htm). ACM includes deaths from all causes, whereas the CVM was coded using the International Classification of Diseases, Tenth Revision codes I00-I09, I11, I13 and I20-51. |
| Age | Divided into three groups: 20-40 years old, 41-60  years old, >60 years old |
| Gender | Male and Female |
| Race | Mexican American, Non-Hispanic Black, Non-Hispanic White, Other Race |
| Educational level | Below high school, High School, or above |
| Marital status | Yes: Married/Living with partner |
| PIR | Poor: <1.3; Not Poor:>=1.3 |
| Obesity | Yes: BMI>=30 |
| Smoking | Smoking status was grouped into never smoker (defined as <100 cigarettes in a lifetime), current smoker (defined as ≥100 cigarettes in a lifetime), and former smoker (defined as ≥100 cigarettes and had quit smoking) |
| Drinking | heavy drinking (≥4 drinks/day for men, ≥3 drinks/day for women, or ≥5 days of drinking in a month),  moderate drinking (≥3 drinks/day for men, ≥2 drinks/day for women, or ≥2 days of drinking in a month),  mild drinking (≤2 drinks/day for men, ≤1 drink/day for women, and ≥12 drinks in a year),  and never-drinking (total number of drinks in a year <12, and dietary alcohol content of 0%) |
| Diabetes | Diabetes was defined as a history of previous diabetes, HbA1c level ≥6.5%, or fasting blood glucose level ≥126 mg/dL |
| Hypertension | The diagnostic criteria consist of self-reported hypertension history, the utilization of antihypertensive medication, a systolic blood pressure (SBP) ≥ 140mmHg, or a diastolic blood pressure (DBP) ≥ 90mmHg |

PIR, poverty income ratio; CDAI, composite dietary antioxidant index.

**Table S2.** Baseline characteristics of all participants according to the CDAI.

| **Characteristic** | **Overall**, N = 30,976 (100%) | **Q1**, N = 8,941 (25%) | **Q2**, N = 7,774 (25%) | **Q3**, N = 7,310 (25%) | **Q4**, N = 6,951 (25%) | **P Value** |
| --- | --- | --- | --- | --- | --- | --- |
| **Age (%)** |  |  |  |  |  | **<0.001** |
| *20-40* | 10,811 (37%) | 2,867 (37%) | 2,661 (37%) | 2,592 (36%) | 2,691 (39%) |  |
| *41-60* | 10,290 (38%) | 2,841 (35%) | 2,487 (36%) | 2,478 (40%) | 2,484 (40%) |  |
| *>60* | 9,875 (25%) | 3,233 (28%) | 2,626 (27%) | 2,240 (25%) | 1,776 (20%) |  |
| **Gender (%)** |  |  |  |  |  | **<0.001** |
| *Male* | 15,051 (48%) | 3,023 (31%) | 3,369 (41%) | 3,883 (51%) | 4,776 (70%) |  |
| *Female* | 15,925 (52%) | 5,918 (69%) | 4,405 (59%) | 3,427 (49%) | 2,175 (30%) |  |
| **Race (%)** |  |  |  |  |  | **<0.001** |
| *Non-Hispanic White* | 13,614 (69%) | 3,545 (64%) | 3,408 (69%) | 3,365 (71%) | 3,296 (72%) |  |
| *Non-Hispanic Black* | 6,659 (11%) | 2,416 (15%) | 1,621 (11%) | 1,388 (9.1%) | 1,234 (8.1%) |  |
| *Other* | 5,952 (12%) | 1,660 (13%) | 1,523 (13%) | 1,432 (12%) | 1,337 (12%) |  |
| *Mexican American* | 4,751 (8.1%) | 1,320 (8.1%) | 1,222 (7.9%) | 1,125 (7.9%) | 1,084 (8.4%) |  |
| **Married/live with partner (%)** |  |  |  |  |  | **<0.001** |
| *No* | 12,334 (36%) | 4,154 (43%) | 3,076 (35%) | 2,628 (32%) | 2,476 (32%) |  |
| *Yes* | 18,642 (64%) | 4,787 (57%) | 4,698 (65%) | 4,682 (68%) | 4,475 (68%) |  |
| **Education level (%)** |  |  |  |  |  | **<0.001** |
| *Below high school* | 7,247 (15%) | 2,818 (22%) | 1,835 (15%) | 1,407 (12%) | 1,187 (11%) |  |
| *High School or above* | 23,729 (85%) | 6,123 (78%) | 5,939 (85%) | 5,903 (88%) | 5,764 (89%) |  |
| **PIR (%)** |  |  |  |  |  | **<0.001** |
| *Not Poor* | 19,850 (80%) | 4,978 (71%) | 5,015 (80%) | 4,990 (84%) | 4,867 (84%) |  |
| *poor* | 8,672 (20%) | 3,184 (29%) | 2,146 (20%) | 1,725 (16%) | 1,617 (16%) |  |
| **Obesity (%)** |  |  |  |  |  | **<0.001** |
| *No* | 18,774 (62%) | 5,100 (59%) | 4,591 (61%) | 4,542 (63%) | 4,541 (66%) |  |
| *Yes* | 11,937 (38%) | 3,733 (41%) | 3,122 (39%) | 2,714 (37%) | 2,368 (34%) |  |
| **Smoking (%)** |  |  |  |  |  | **<0.001** |
| *Never* | 17,042 (55%) | 4,727 (51%) | 4,417 (57%) | 4,097 (56%) | 3,801 (56%) |  |
| *Former* | 7,697 (25%) | 1,934 (21%) | 1,908 (24%) | 1,958 (28%) | 1,897 (28%) |  |
| *Current* | 6,237 (20%) | 2,280 (28%) | 1,449 (18%) | 1,255 (16%) | 1,253 (16%) |  |
| **Drinking (%)** |  |  |  |  |  | **<0.001** |
| *former* | 4,973 (13%) | 1,679 (16%) | 1,251 (13%) | 1,096 (13%) | 947 (12%) |  |
| *heavy* | 5,950 (21%) | 1,655 (22%) | 1,409 (20%) | 1,396 (20%) | 1,490 (22%) |  |
| *mild* | 10,157 (37%) | 2,354 (29%) | 2,592 (37%) | 2,619 (40%) | 2,592 (42%) |  |
| *moderate* | 4,684 (18%) | 1,295 (18%) | 1,224 (19%) | 1,115 (17%) | 1,050 (17%) |  |
| *never* | 4,256 (11%) | 1,619 (15%) | 1,071 (11%) | 894 (9.6%) | 672 (7.7%) |  |
| **Hypertension (%)** |  |  |  |  |  | **<0.001** |
| *No* | 17,298 (61%) | 4,513 (58%) | 4,276 (61%) | 4,282 (63%) | 4,227 (65%) |  |
| *Yes* | 13,347 (39%) | 4,319 (42%) | 3,409 (39%) | 2,957 (37%) | 2,662 (35%) |  |
| **Diabetes (%)** |  |  |  |  |  | **<0.001** |
| *No* | 11,632 (75%) | 3,195 (71%) | 2,915 (74%) | 2,864 (77%) | 2,658 (78%) |  |
| *Yes* | 5,477 (25%) | 1,906 (29%) | 1,416 (26%) | 1,174 (23%) | 981 (22%) |  |
| **CDAI (mean (SD))** | 1.28 (4.83) | -3.72 (1.38) | -0.66 (0.70) | 1.96 (0.87) | 7.55 (4.65) | **<0.001** |

Mean (SD) for continuous variables: the P value was calculated by the weighted linear regression model.

Percentages (weighted N, %) for categorical variables: the P value was calculated by the weighted chi-square test.

Abbreviation: CDAI, composite dietary antioxidant index; PIR, poverty income ratio.

**Table S3.** Baseline characteristics of participants with suicidal ideation, according to the CDAI.

| **Characteristic** | **Overall**, N = 1,154 (100%) | **Q1**, N = 458 (37%) | **Q2**, N = 258 (22%) | **Q3**, N = 226 (20%) | **Q4**, N = 212 (21%) | **P Value** |
| --- | --- | --- | --- | --- | --- | --- |
| **Age (%)** |  |  |  |  |  | 0.061 |
| *20-40* | 360 (35%) | 137 (36%) | 72 (32%) | 82 (40%) | 69 (34%) |  |
| *41-60* | 460 (42%) | 179 (43%) | 101 (38%) | 87 (37%) | 93 (50%) |  |
| *>60* | 334 (23%) | 142 (21%) | 85 (30%) | 57 (23%) | 50 (16%) |  |
| **Gender (%)** |  |  |  |  |  | **<0.001** |
| *Male* | 522 (46%) | 158 (33%) | 102 (36%) | 130 (61%) | 132 (64%) |  |
| *Female* | 632 (54%) | 300 (67%) | 156 (64%) | 96 (39%) | 80 (36%) |  |
| **Race (%)** |  |  |  |  |  | 0.539 |
| *Non-Hispanic White* | 457 (61%) | 179 (59%) | 94 (58%) | 87 (60%) | 97 (68%) |  |
| *Other* | 279 (18%) | 110 (18%) | 65 (20%) | 59 (18%) | 45 (14%) |  |
| *Non-Hispanic Black* | 221 (12%) | 95 (14%) | 43 (11%) | 43 (13%) | 40 (9.8%) |  |
| *Mexican American* | 197 (9.3%) | 74 (9.1%) | 56 (11%) | 37 (9.7%) | 30 (7.5%) |  |
| **Married/live with partner (%)** |  |  |  |  |  | 0.177 |
| *No* | 638 (53%) | 275 (59%) | 137 (50%) | 112 (50%) | 114 (50%) |  |
| *Yes* | 516 (47%) | 183 (41%) | 121 (50%) | 114 (50%) | 98 (50%) |  |
| **Education level (%)** |  |  |  |  |  | **<0.001** |
| *Below high school* | 443 (27%) | 204 (33%) | 109 (30%) | 75 (26%) | 55 (15%) |  |
| *High School or above* | 711 (73%) | 254 (67%) | 149 (70%) | 151 (74%) | 157 (85%) |  |
| **PIR (%)** |  |  |  |  |  | **0.005** |
| *Not Poor* | 524 (60%) | 184 (53%) | 110 (57%) | 115 (66%) | 115 (70%) |  |
| *poor* | 532 (40%) | 233 (47%) | 129 (43%) | 86 (34%) | 84 (30%) |  |
| **Obesity (%)** |  |  |  |  |  | 0.463 |
| *No* | 641 (57%) | 256 (55%) | 134 (53%) | 121 (57%) | 130 (62%) |  |
| *Yes* | 492 (43%) | 191 (45%) | 118 (47%) | 101 (43%) | 82 (38%) |  |
| **Smoking (%)** |  |  |  |  |  | **0.018** |
| *Never* | 495 (44%) | 186 (42%) | 113 (42%) | 100 (42%) | 96 (50%) |  |
| *Former* | 280 (24%) | 97 (18%) | 60 (25%) | 58 (29%) | 65 (27%) |  |
| *Current* | 379 (33%) | 175 (40%) | 85 (33%) | 68 (29%) | 51 (23%) |  |
| **Drinking (%)** |  |  |  |  |  | 0.056 |
| *former* | 245 (19%) | 92 (17%) | 65 (21%) | 47 (18%) | 41 (19%) |  |
| *heavy* | 282 (26%) | 118 (30%) | 60 (25%) | 52 (24%) | 52 (23%) |  |
| *mild* | 274 (28%) | 96 (21%) | 58 (29%) | 61 (31%) | 59 (35%) |  |
| *moderate* | 152 (15%) | 48 (13%) | 37 (15%) | 36 (19%) | 31 (14%) |  |
| *never* | 156 (12%) | 85 (18%) | 30 (10%) | 23 (7.9%) | 18 (8.6%) |  |
| **Hypertension (%)** |  |  |  |  |  | 0.137 |
| *No* | 560 (55%) | 206 (54%) | 115 (49%) | 122 (54%) | 117 (62%) |  |
| *Yes* | 579 (45%) | 245 (46%) | 138 (51%) | 102 (46%) | 94 (38%) |  |
| **Diabetes (%)** |  |  |  |  |  | 0.565 |
| *No* | 378 (67%) | 149 (64%) | 83 (66%) | 79 (71%) | 67 (69%) |  |
| *Yes* | 280 (33%) | 117 (36%) | 73 (34%) | 49 (29%) | 41 (31%) |  |
| **CDAI (mean (SD))** | 0.40 (5.13) | -4.02 (1.52) | -0.74 (0.68) | 1.97 (0.87) | 7.89 (5.04) | **<0.001** |

Mean (SD) for continuous variables: the P value was calculated by the weighted linear regression model.

Percentages (weighted N, %) for categorical variables: the P value was calculated by the weighted chi-square test.

Abbreviation: CDAI, composite dietary antioxidant index; PIR, poverty income ratio.

**Table S4.** Baseline characteristics of participants without suicidal ideation, according to the CDAI.

| **Characteristic** | **Overall**, N = 29,822 (100%) | **Q1**, N = 8,483 (25%) | **Q2**, N = 7,516 (25%) | **Q3**, N = 7,084 (25%) | **Q4**, N = 6,739 (25%) | **P Value** |
| --- | --- | --- | --- | --- | --- | --- |
| **Age (%)** |  |  |  |  |  | **<0.001** |
| *20-40* | 10,451 (37%) | 2,730 (37%) | 2,589 (37%) | 2,510 (35%) | 2,622 (39%) |  |
| *41-60* | 9,830 (38%) | 2,662 (35%) | 2,386 (36%) | 2,391 (40%) | 2,391 (40%) |  |
| *>60* | 9,541 (25%) | 3,091 (28%) | 2,541 (27%) | 2,183 (25%) | 1,726 (21%) |  |
| **Gender (%)** |  |  |  |  |  | **<0.001** |
| *Male* | 14,529 (48%) | 2,865 (31%) | 3,267 (41%) | 3,753 (51%) | 4,644 (70%) |  |
| *Female* | 15,293 (52%) | 5,618 (69%) | 4,249 (59%) | 3,331 (49%) | 2,095 (30%) |  |
| **Race (%)** |  |  |  |  |  | **<0.001** |
| *Non-Hispanic White* | 13,157 (69%) | 3,366 (64%) | 3,314 (69%) | 3,278 (71%) | 3,199 (72%) |  |
| *Non-Hispanic Black* | 6,438 (11%) | 2,321 (15%) | 1,578 (11%) | 1,345 (9.0%) | 1,194 (8.1%) |  |
| *Other* | 5,673 (12%) | 1,550 (12%) | 1,458 (12%) | 1,373 (12%) | 1,292 (11%) |  |
| *Mexican American* | 4,554 (8.0%) | 1,246 (8.0%) | 1,166 (7.8%) | 1,088 (7.9%) | 1,054 (8.4%) |  |
| **Married/live with partner (%)** |  |  |  |  |  | **<0.001** |
| *No* | 11,696 (35%) | 3,879 (42%) | 2,939 (34%) | 2,516 (32%) | 2,362 (32%) |  |
| *Yes* | 18,126 (65%) | 4,604 (58%) | 4,577 (66%) | 4,568 (68%) | 4,377 (68%) |  |
| **Education level (%)** |  |  |  |  |  | **<0.001** |
| *Below high school* | 6,804 (14%) | 2,614 (21%) | 1,726 (14%) | 1,332 (12%) | 1,132 (11%) |  |
| *High School or above* | 23,018 (86%) | 5,869 (79%) | 5,790 (86%) | 5,752 (88%) | 5,607 (89%) |  |
| **PIR (%)** |  |  |  |  |  | **<0.001** |
| *Not Poor* | 19,326 (81%) | 4,794 (72%) | 4,905 (81%) | 4,875 (85%) | 4,752 (84%) |  |
| *poor* | 8,140 (19%) | 2,951 (28%) | 2,017 (19%) | 1,639 (15%) | 1,533 (16%) |  |
| **Obesity (%)** |  |  |  |  |  | **<0.001** |
| *No* | 18,133 (62%) | 4,844 (59%) | 4,457 (61%) | 4,421 (63%) | 4,411 (66%) |  |
| *Yes* | 11,445 (38%) | 3,542 (41%) | 3,004 (39%) | 2,613 (37%) | 2,286 (34%) |  |
| **Smoking (%)** |  |  |  |  |  | **<0.001** |
| *Never* | 16,547 (55%) | 4,541 (51%) | 4,304 (58%) | 3,997 (57%) | 3,705 (56%) |  |
| *Former* | 7,417 (25%) | 1,837 (21%) | 1,848 (24%) | 1,900 (28%) | 1,832 (28%) |  |
| *Current* | 5,858 (19%) | 2,105 (27%) | 1,364 (18%) | 1,187 (16%) | 1,202 (16%) |  |
| **Drinking (%)** |  |  |  |  |  | **<0.001** |
| *former* | 4,728 (13%) | 1,587 (16%) | 1,186 (13%) | 1,049 (13%) | 906 (11%) |  |
| *heavy* | 5,668 (21%) | 1,537 (22%) | 1,349 (20%) | 1,344 (19%) | 1,438 (22%) |  |
| *mild* | 9,883 (37%) | 2,258 (29%) | 2,534 (38%) | 2,558 (41%) | 2,533 (42%) |  |
| *moderate* | 4,532 (18%) | 1,247 (18%) | 1,187 (19%) | 1,079 (17%) | 1,019 (17%) |  |
| *never* | 4,100 (11%) | 1,534 (15%) | 1,041 (11%) | 871 (9.7%) | 654 (7.6%) |  |
| **Hypertension (%)** |  |  |  |  |  | **<0.001** |
| *No* | 16,738 (62%) | 4,307 (58%) | 4,161 (61%) | 4,160 (63%) | 4,110 (65%) |  |
| *Yes* | 12,768 (38%) | 4,074 (42%) | 3,271 (39%) | 2,855 (37%) | 2,568 (35%) |  |
| **Diabetes (%)** |  |  |  |  |  | **<0.001** |
| *No* | 11,254 (75%) | 3,046 (71%) | 2,832 (75%) | 2,785 (77%) | 2,591 (79%) |  |
| *Yes* | 5,197 (25%) | 1,789 (29%) | 1,343 (25%) | 1,125 (23%) | 940 (21%) |  |
| **CDAI (mean (SD))** | 1.31 (4.82) | -3.71 (1.37) | -0.66 (0.70) | 1.96 (0.87) | 7.54 (4.64) | **<0.001** |

Mean (SD) for continuous variables: the P value was calculated by the weighted linear regression model.

Percentages (weighted N, %) for categorical variables: the P value was calculated by the weighted chi-square test.

Abbreviation: CDAI, composite dietary antioxidant index; PIR, poverty income ratio.

**
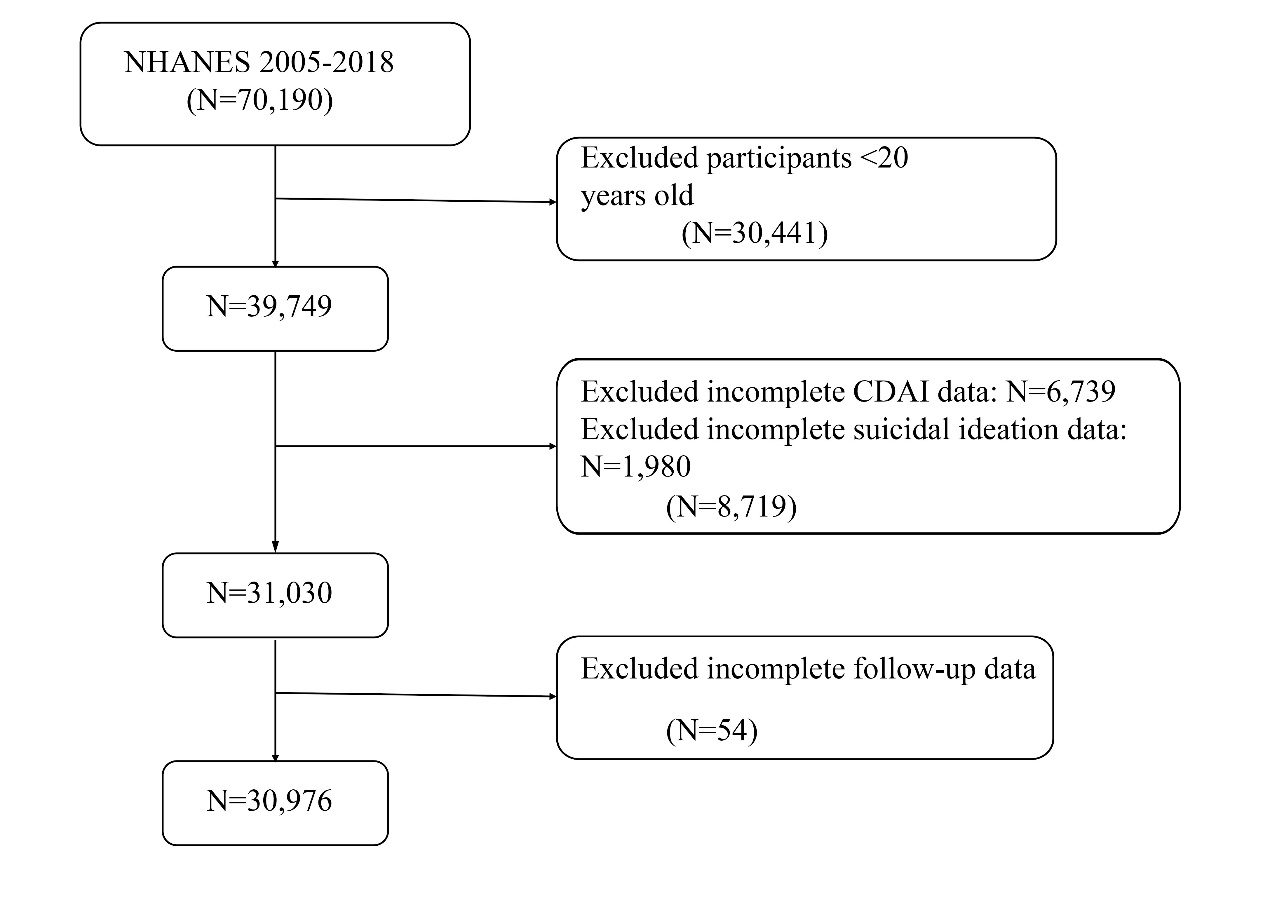
**

**Figure S1.** A flow diagram of eligible participant selection in the National Health and Nutrition Examination Survey.

**
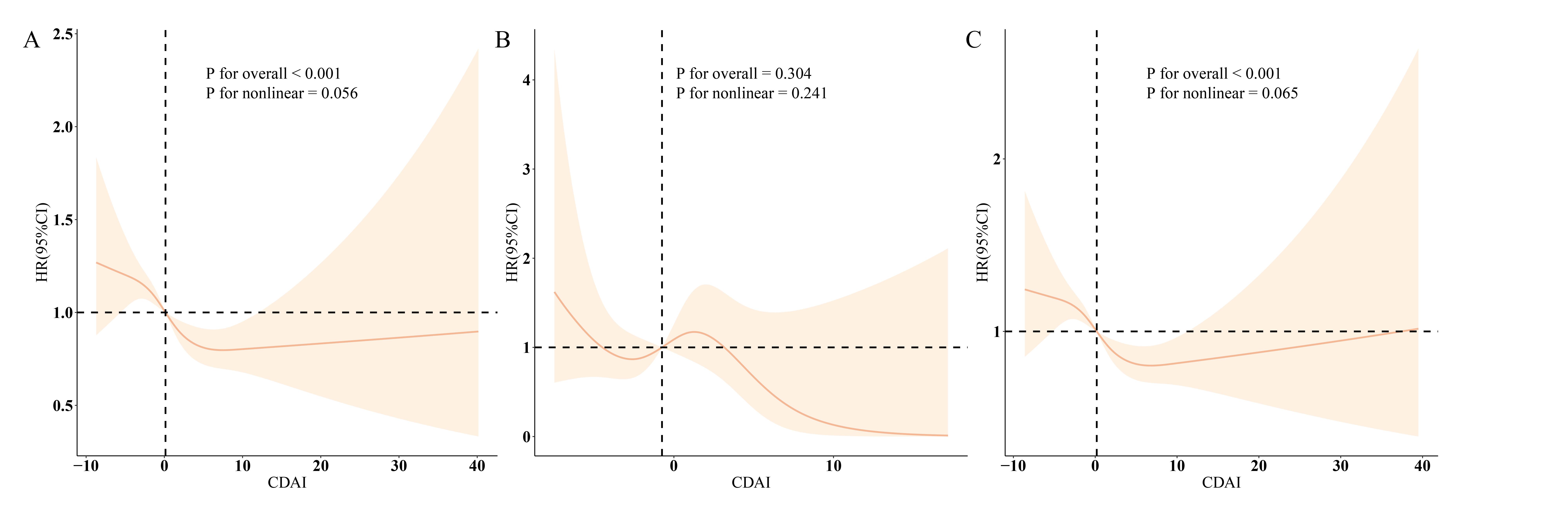
**

**Figure S2.** Restricted cubic spline curves for the association between CDAI and all-cause mortality in (A) total participants, (B) participants with suicidal ideation, and (C) participants without suicidal ideation.

HR (solid lines) and 95% confidence levels (shaded areas) were adjusted for age, gender, education level, marital status, PIR, race, obesity, smoking, drinking, hypertension, and diabetes.

Abbreviation: PIR, poverty income ratio; CDAI, composite dietary antioxidant index; HR, hazard ratio; CI, confidence interval.

**
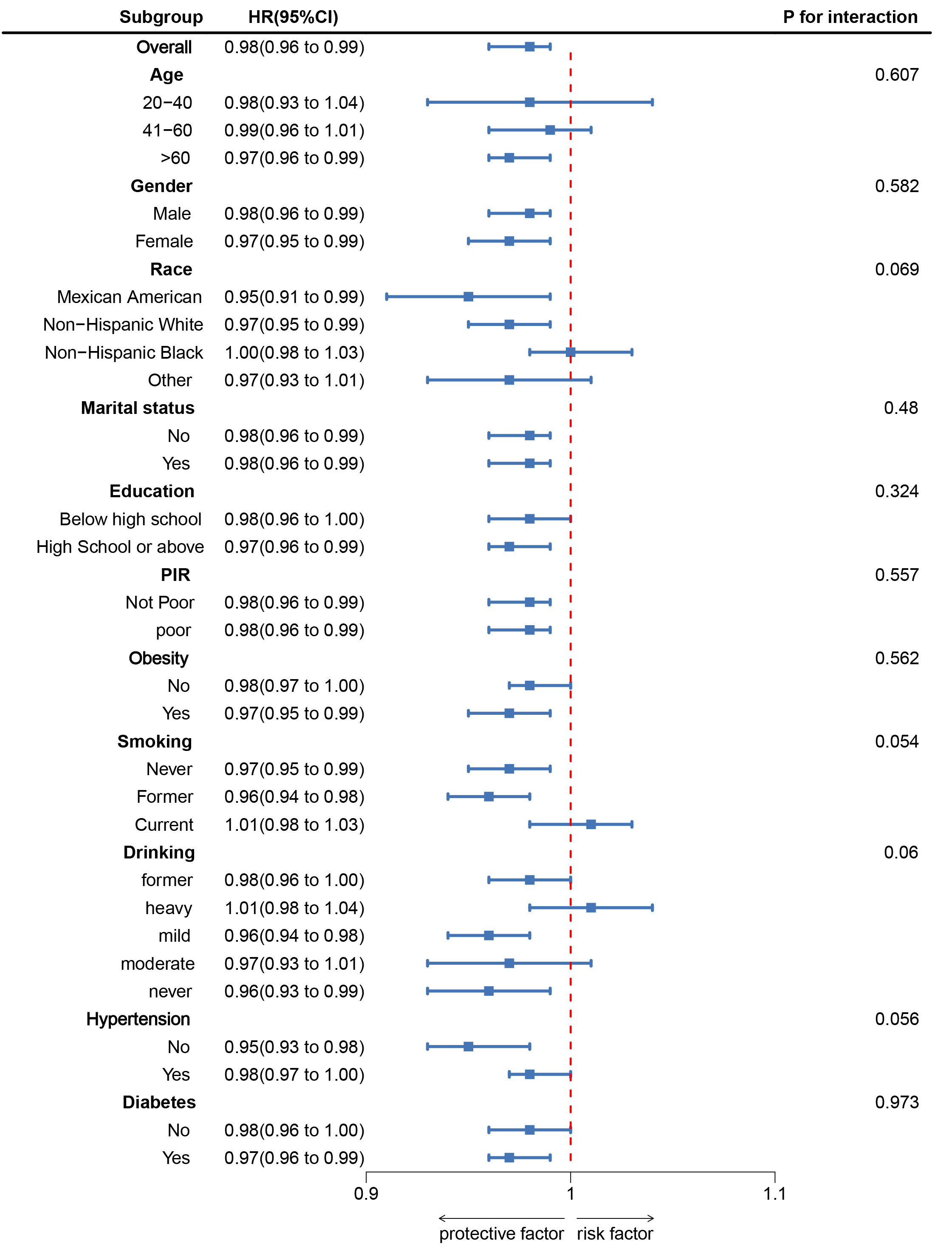
**

**Figure S3.** Subgroup analysis of the association between CDAI and all-cause mortality in all the participants. Adjusted for age, gender, education level, marital status, PIR, race, obesity, smoking, drinking, hypertension, and diabetes. HR: Hazard ratio, CI: confidence interval.

**
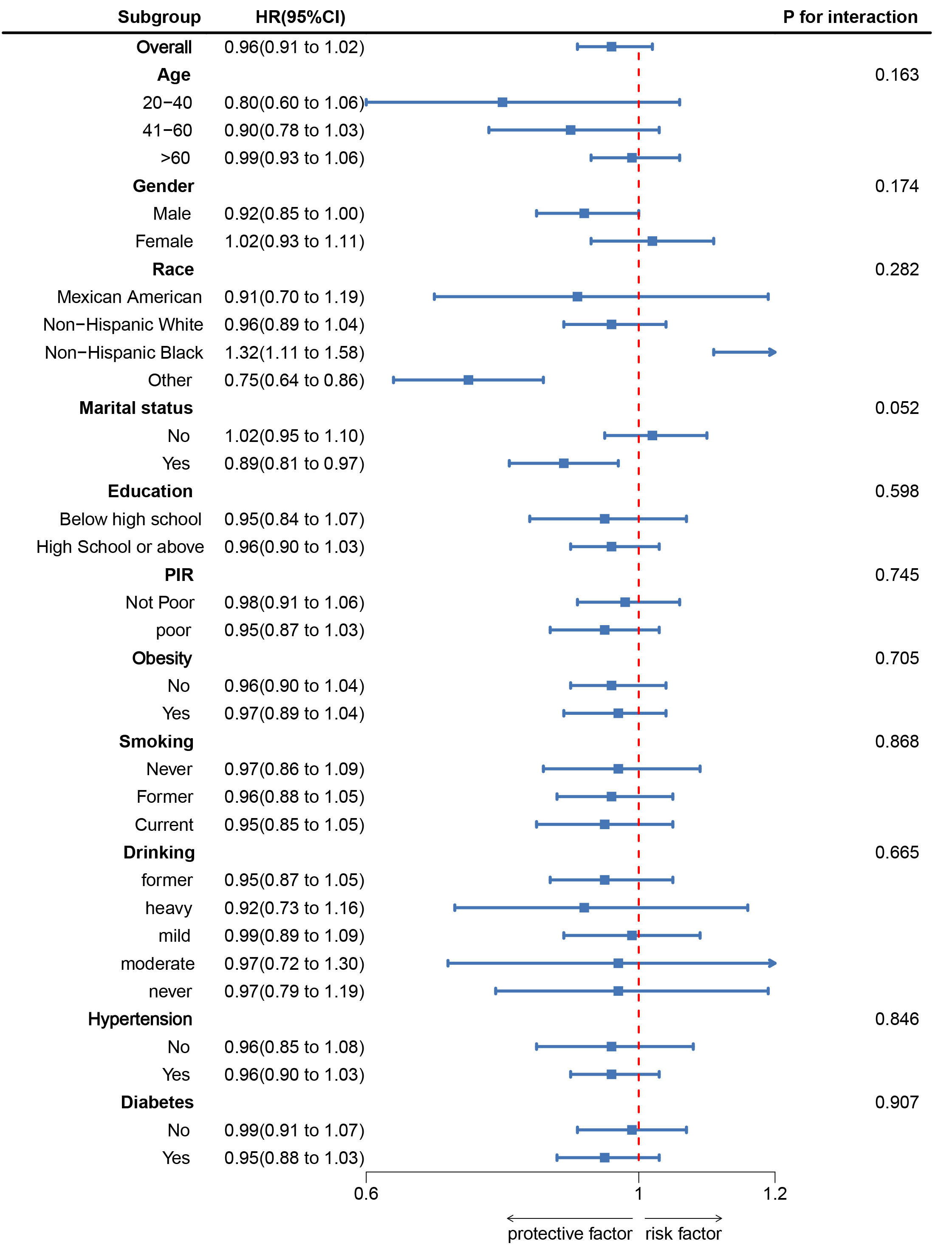
**

**Figure S4.** Subgroup analysis of the association between CDAI and all-cause mortality in participants with suicidal ideation. Adjusted for age, gender, education level, marital status, PIR, race, obesity, smoking, drinking, hypertension, and diabetes. HR: Hazard ratio, CI: confidence interval.

**
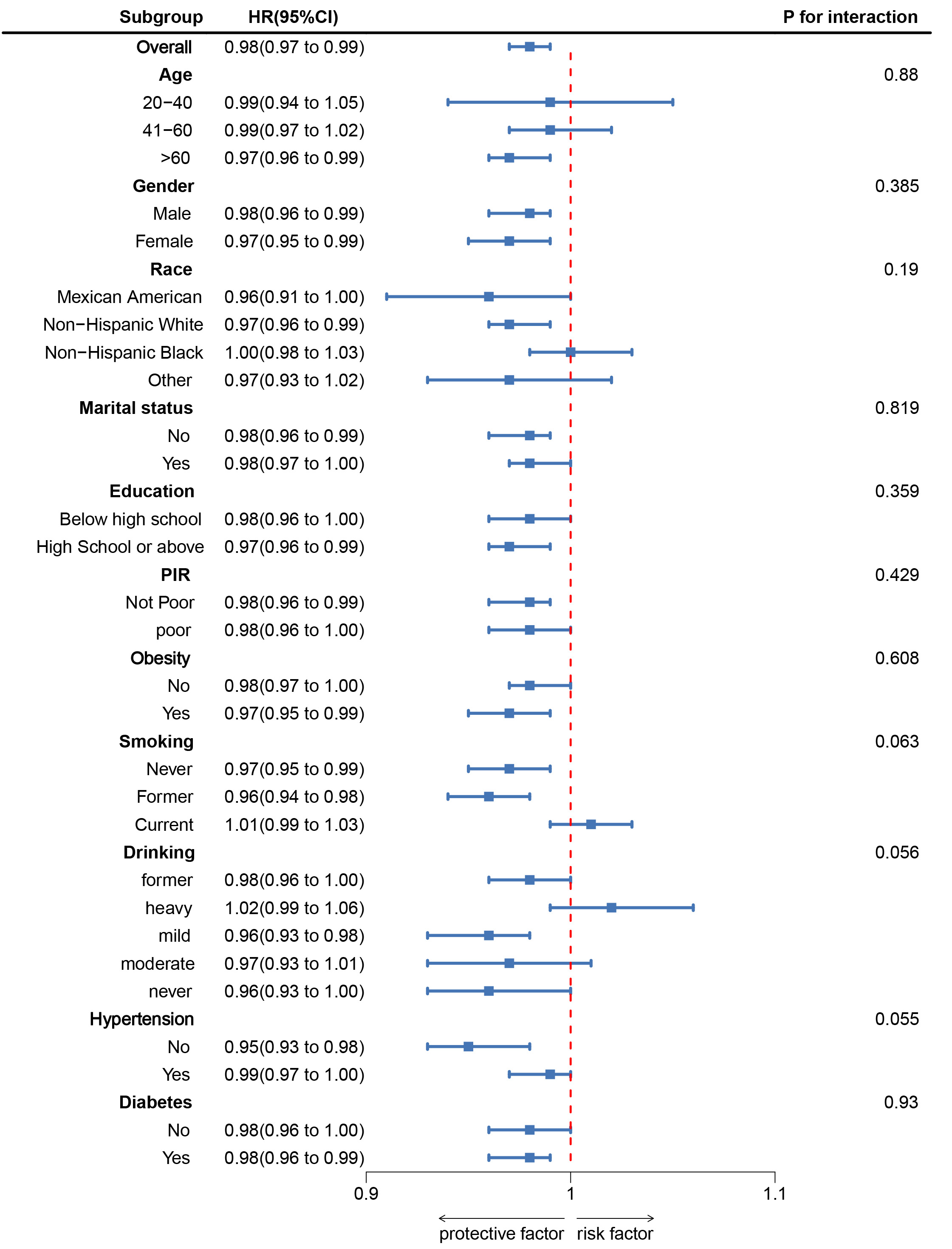
**

**Figure S5.** Subgroup analysis of the association between CDAI and all-cause mortality in participants without suicidal ideation. Adjusted for age, gender, education level, marital status, PIR, race, obesity, smoking, drinking, hypertension, and diabetes. HR: Hazard ratio, CI: confidence interval.
